# Aquaporin 4: a player in cerebral edema and neuroinflammation

**DOI:** 10.1186/1742-2094-9-279

**Published:** 2012-12-27

**Authors:** Andrew M Fukuda, Jerome Badaut

**Affiliations:** 1 Departments of Physiology, Loma Linda University School of Medicine, Loma Linda, CA, 92354, USA; 2 Departments of Pediatrics, Loma Linda University School of Medicine, Loma Linda, CA, 92354, USA

**Keywords:** Aquaporin, Astrocyte, Blood brain barrier, Stroke, Traumatic brain injury, Neuroinflammation

## Abstract

Neuroinflammation is a common pathological event observed in many different brain diseases, frequently associated with blood brain barrier (BBB) dysfunction and followed by cerebral edema. Neuroinflammation is characterized with microglia activation and astrogliosis, which is a hypertrophy of the astrocytes. Astrocytes express aquaporin 4, the water channel protein, involved in water homeostasis and edema formation. Aside from its function in water homeostasis, recent studies started to show possible interrelations between aquaporin 4 and neuroinflammation. In this review the roles of aquaporin 4 in neuroinflammation associated with BBB disruption and cerebral edema will be discussed with recent studies in the field.

## 

The purpose of this review is not to discuss neuroinflammation mechanisms or an extensive review of aquaporin 4 (AQP4), for there are numerous reviews covering these two topics independently. Rather, we address the question whether AQP4 is a common player between edema and neuroinflammation by reviewing the recent literature in the field. In recent years, AQP4 has been associated with neuroinflammation in chronic and acute brain diseases [1-12]. Since AQP4 is mostly expressed on the astrocytes, and because neuroinflammation is characterized by both phenotypic changes of resting astrocytes to astrogliosis and microglial activation, we believe that tackling this question can lead to a better understanding of many brain diseases.

## General introduction

### Aquaporin 4 - overview

**Figure 1:**
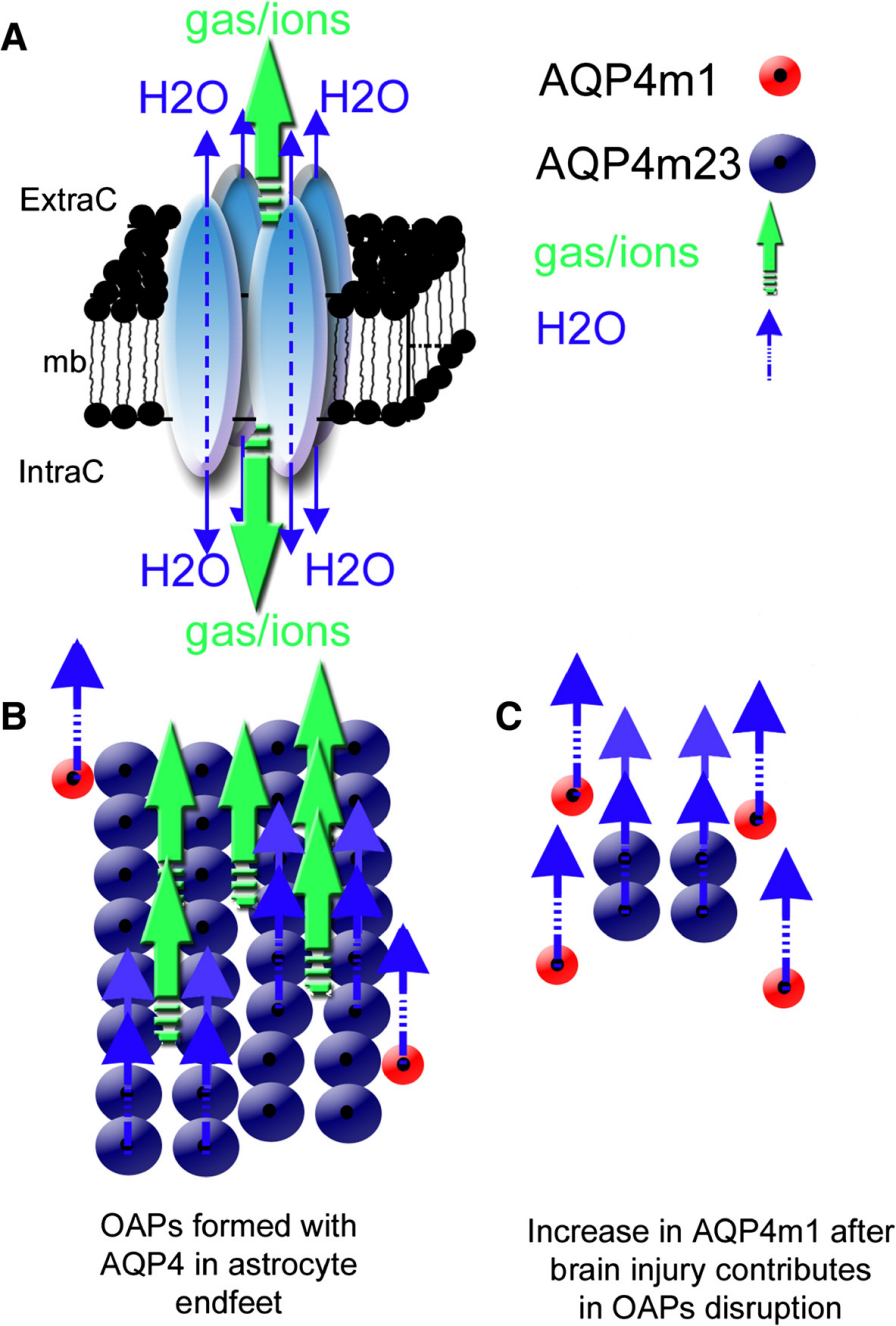
**Structural organization of AQP4 in the astrocyte membrane. **(**A**) Schematic drawing of the AQP4 homo-tetramer assembly within the lipid membrane from a lateral view resulting in a central pore permeable to cations and gases (green arrows) [16]. Each individual aquaporin facilitates bi-directional water movement that is dependent on the osmotic gradient (blue arrows) (modified from Badaut *et al*. [57]). (**B**) In normal brain, association between AQP4-m1 (red circles) and AQP4-m23 (blue circles) isoforms contribute to form orthogonal array of particles (OAPs). Higher expression of AQP4-m23 contributes to the formation of large OAPs, and should facilitate the gas, ion (green arrows), and water diffusion (water arrows) through the astrocyte membrane. (**C**) In brain injury, increase of AQP4-m1 [19] should contribute to disruption of OAPs (modified from Badaut *et al*. [57]). Changes in OAP size may decrease the number of central pore and possibly affect not only water movement but also the ion and gas movements.

Aquaporin (AQP) is a family of water channel protein ubiquitously expressed in various cell types and organisms [13]. The aquaporin family exhibits a common structure with six membrane spanning alpha helical domains, a consensus motif composed of Asparagine-Proline-Alanine (NPA) constituting part of the pore, and an approximate molecular weight of 30 kDa [14]. AQP4 is the most abundant AQP found in the primate and rodent brains, mainly in the perivascular astrocyte endfeet [13]. AQP4 is assembled in homo-tetramers where each individual aquaporin represents a water channel (Figure 1A) [15]. The assemblage of four molecules of AQP4 forms a central pore, through which water, cations, and gases such as CO_2_ flow [16]. Interestingly, AQP4 proteins are major constituents of a higher structural arrangement within astrocyte endfoot named the orthogonal arrays of particles (OAPs) observed using electron microscopy after cryo-fracture preparation [17] (Figure 1B). The size of the OAPs is determined by the ratio between the two main isoforms of AQP4: the long (AQP4-m1) and short (AQP4-m23) splice variants (Figure 1B) [17]. The AQP4-m23 isoform stabilizes the OAP structure [17,18]. The exact functional roles of the OAPs remain unknown both in normal and pathological brains. An increase in the AQP4 m1 variant disrupts the structure of the OAPs [17,18], which is observed in stroke [19,20] and parallels blood–brain barrier (BBB) disruption. The role of AQP4 within the perivascular space and in BBB structure is still a matter of discussion and unresolved [21,22]. Interestingly, OAPs were proposed to play a role in potassium buffering [23,24]. Strengthening this hypothesis, AQP4 found in the astrocyte endfeet facing cerebral blood vessels co-localized with the potassium channel, K_ir_4.1 [25,26]. AQP4 knockout mice (AQP4^−/−^) showed a delay in potassium reuptake suggesting that AQP4 has a role in potassium homeostasis in facilitating water diffusion along the potassium gradient during brain activity [27]. Not only involved in water movement, AQP4 may also contribute to cell adhesion [28] and migration [29]. These data underline the diversity and complexity in AQP4 functions, and the subsequent sections will show another possible function of AQP4 in the process of neuroinflammation.

### Microglia, astrocytes, and AQP4 in the context of neuroinflammation

Neuroinflammation is largely described in the acute phase after brain injury, along with edema, as well as in chronic brain diseases like multiple sclerosis. The term, ‘neuroinflammation’, encompasses several molecular and cellular modifications without a clear, unified definition amongst the various brain diseases and injuries. However, it is important to realize that neuroinflammation is distinct from peripheral inflammation [30-32], even if they share some of the same molecular players, particularly due to microglia and astrocytes, which are cells specific to the CNS. Since the inflammatory process may differ from organ to organ [31], Graeber and colleagues recently drew the attention to the potential danger of using the two terminologies ‘neuroinflammation’ and ‘inflammation’ interchangeably.

Although microglia is the cell type considered to be primarily responsible for the innate immune response in the CNS [33,34], it is premature to conclude that a decrease in microglial activation is the only evidence needed for the treatment of neuroinflammation with anti-inflammatory drugs [30]. Activation status for both astrocytes and microglia, along with secretion of cytokines and chemokines, should be considered for neuroinflammation. Microglial activation is characterized by morphological changes in which the usually ramified microglia becomes round with no ramifications [33]. There seems to be a dual role for microglial activation in which acute activation is beneficial [35] but a chronic one is detrimental [33,36]. In fact, activated microglia is responsible for producing pro-inflammatory cytokines such as IL-1β, IL-6, TNFα and also anti-inflammatory cytokines such as IL-4, IL-10 and TFGβ [37].

Activated astrocytes also play a key role in neuroinflammation with their involvement in astrogliosis, although whether astrogliosis is beneficial or detrimental seem to depend on the situation [38], much like microglial activation. The process of astrogliosis includes the hyptertrophy of astrocytes with different morphological fates depending on the severity of the injury [39]. The absence of AQP4 in astrocytic endfeet may lead to decreased hypertrophy of astrocytes due to decreased water entry and migration toward the site of the injury [29,40]. Like microglia, activated astrocytes contribute to the secretion of chemokines and cytokines (see examples above), possibly involved in BBB disruption and vasogenic edema. Interestingly, AQP4 is upregulated in the vasogenic edema resolution phase visualized by normalization of magnetic resonance (MR) signals in several disease models [2,41]. Furthermore, AQP4 has also been reported to be present in reactive microglia after intranigral injection of lipopolysaccharide (LPS) in rats, although the functional significance of this *de novo* microglial AQP4 expression is unknown [42]. These changes in AQP4 during the inflammatory process suggest changes in water movement related to neuroinflammation.

## AQP4 and neuroinflammation in autoimmune diseases

### Experimental autoimmune encephalomyelitis (EAE) and AQP4

Recent data in a model of experimental autoimmune encephalomyelitis (EAE) in which homogenized guinea-pig whole spinal cord was injected into rats showed upregulation of AQP4 starting at 10 days until the onset and peak of cerebellar enlargement. At these timepoints, significantly positive correlation was observed between AQP4 and BBB disruption in the cerebellum, associated with a decrease of tight junction proteins such as occludin [7]. This detrimental role of AQP4 in EAE is supported by a less severe clinical and tissue inflammation score after EAE and LPS-injection in AQP4^−/−^ mice than WT animals [1]. This is most likely the cause of reduced production of the pro-inflammatory cytokines, TNFα and IL-6, observed in AQP4^−/−^ mice astrocyte cultures [1].

AQP4^−/−^ mice studies have also suggested that AQP4 could be contributing to the production of CD4+ and CD25+ T regulator cells; and lack of AQP4 may be disrupting the immunosuppressive regulators in Parkinson’s disease, leading to increased microglial activation and a worse outcome due to more dopaminergic neuronal loss after induction of 1-methyl-4-phenyl-1,2,3,6-tetrahydropyridine [5]. Interestingly, AQP4 expression is present in the spleen, lymph nodes, and thymus, hinting towards a more direct role of AQP4 in systemic immune responses, and perhaps not just confined to neuroinflammation [5].

### Neuromyelitis optica (NMO) and AQP4

The possible link between neuroinflammation and AQP4 was advertised with neuromyelitis optica (NMO), a demyelinating disease. NMO is a pathological condition characterized by abnormal signals most often observed in the spinal cord and optic nerve, and in the form of blindness and paralysis. Interestingly, AQP4 has been identified as the target for NMO-IgG, a unique feature of the disease which differentiates it from multiple sclerosis [43-45], making it a very useful differential diagnostic tool in the clinics. More specifically, there is plausible evidence that NMO-IgG specifically targets AQP4 within the OAP structures, rather than free AQP4 isoforms [6,46,47]. Whether the presence of an autoantibody against AQP4 is the cause of the disease or a collateral consequence of some secondary pathological mechanisms still lacks an unanimous answer, but studies performed where immunoglobulins taken from AQP4 antibody positive NMO patients were administered to rats with EAE showed NMO pathology seen in the clinics [48,49], suggesting that the presence of AQP4 autoantibody in patients already suffering from neuroimmune disease worsens the condition and leads to the NMO pathology observed. Interestingly, several clinical observations have been reported in which patients with myasthenia gravis (MG) also suffer from auto-AQP4-antibody positive NMO simultaneously [50-56]. Thus pointing out the possibility of a common autoimmune origin for both diseases, or the aforementioned worsening effect of the AQP4 autoantibody in patients with pre-existing immune diseases; previously unrecognized because of the lack of knowledge about the NMO IgG auto-AQP4 antibody as a diagnostic tool for NMO. This link could point to the involvement of AQP4 in the peripheral immune system as well.

In summary, these recent data from NMO and AQP4^−/−^ mice models are encouraging to propose that AQP4 is a player in inflammation and neuroinflammation. But considering AQP4 properties as a water channel, its function in these processes are still unclear.

## Neuroinflammation and edema in brain injury: astrocyte AQP4

### BBB breakdown and vasogenic edema

**Figure 2:**
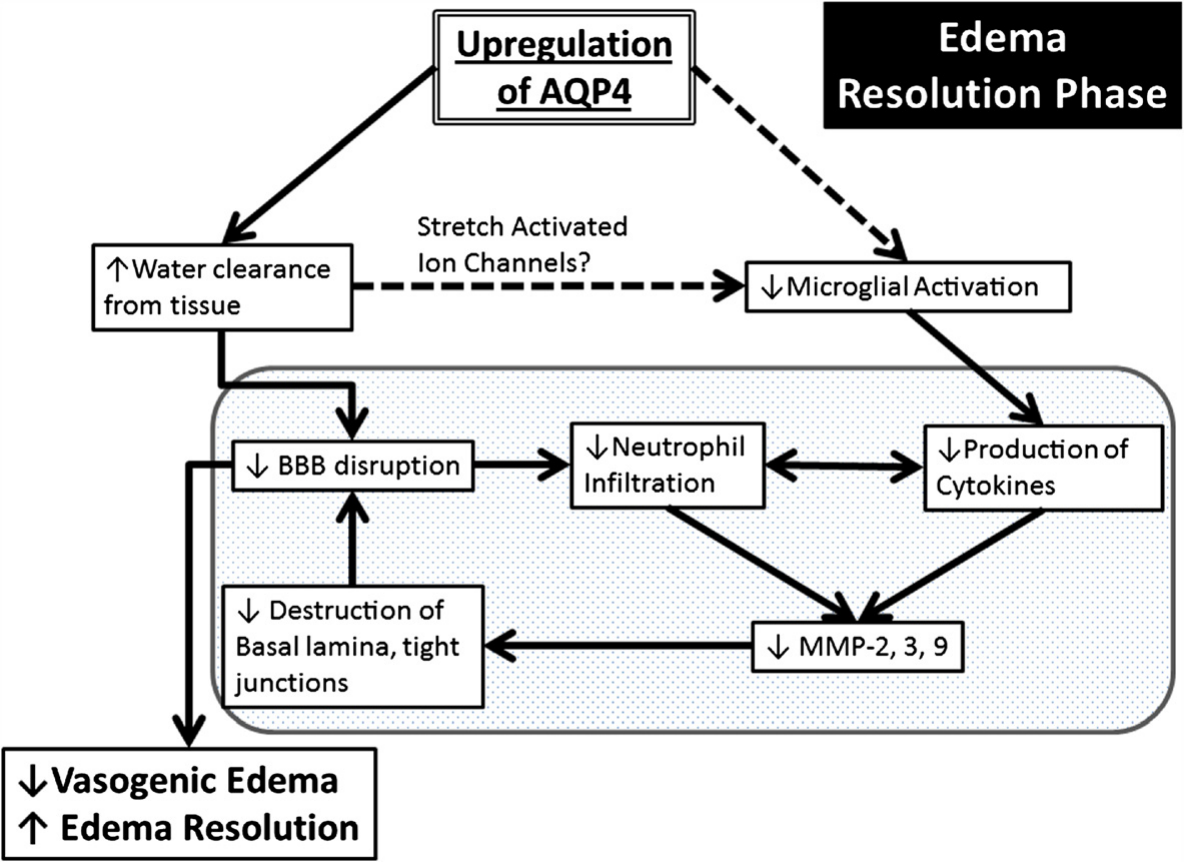
**Schematic summary of a beneficial role AQP4 upregulation plays during the edema resolution phase.** The upregulation of AQP4 causes increased water clearance from the tissue, which in turn causes decreased BBB disruption because of decreased pressure, and there is less neutrophil infiltration and decreased pro-inflammatory cytokines. This cause decreased MMP production [62], which possibly results in less destruction of the basal lamina and tight junctions, causes an even greater decrease of the BBB. In another pathway (dotted lines), the increased water clearance from the tissue and extracellular space causes changes in the osmotic pressure, changing the activation state of the stretch activated ion channels expressed in microglia [86-88], causing less microglial activation, thus causing decreased pro-inflammatory cytokine. The resulting decrease in BBB disruption/permeability leads to decreased vasogenic edema or better edema resolution. Finally, this scheme outlines the potential link between AQP4, edema and neuroinflammation.

AQP4 is one of the key players in edema formation and resolution [57,58] and increase in its expression is observed in reactive astrocytes after injury. Edema is frequently observed in brain injuries and is associated with BBB disruption [57,59]. Compromised BBB integrity leads to plasma protein leakage and extravascular fluid accumulation [57]. The breakdown of the BBB is a complex process partially caused by the activation of matrix metalloproteinases (MMPs), which is part of the neuroinflammatory response [60-62]. Pro-inflammatory cytokines such as IL-1β and TNFα has been shown to produce MMP-9 and MMP-3 in cultured astrocytes and microglia (reviewed in [62]). MMP-9 aggravates vasogenic edema development by degrading the basal lamina located between the astrocytic endfeet and endothelia [62]. Of particular interest is the link of MMP with AQP4; MMP-2 and MMP-9 are known to degrade agrin and MMP-3 degrades dystroglycan [63], two proteins that have a critical role in the maintenance of the OAP [64-67]. So, when MMP are upregulated after a neuroinflammatory response, more AQP4-OAPs will be disorganized, leading to a possible disruption of the BBB and edema. Vasogenic edema development can further damage the endothelia by increased water volume and therefore increased hydrostatic pressure. Thus, if there is decreased BBB disruption, there will be less pro-inflammatory cytokines, MMPs, and edema (Figure 2).

### Magnetic resonance imaging and AQP4

One useful modality in assessing injury severity and outcome in cerebral pathological conditions both in clinics and research is magnetic resonance imaging (MRI). Because MRI detects changes observed via the excitation of water molecules, the presence of the water channel protein, AQP4 in astrocytes suggests a possible involvement in MRI. Diffusion-weighted magnetic resonance imaging (DWI) is widely used as a diagnostic tool in clinical and research settings to assess edematous damage after various brain pathologies from ischemic stroke to various neuroinflammatory diseases [57,68-71]. The apparent diffusion coefficient (ADC) is obtained from DWI and is used to evaluate cerebral changes in clinical and experimental models. A decrease in the ADC is classically associated with a decrease in the extracellular space during cell swelling after brain injury [70]. More recently, ADC changes have been hypothesized to be linked with the level of expression of AQP4. Several experiments have shown increases in AQP4 expression and increased ADC [71] and decreased AQP4 expression with decreased ADC [58,72]. Of note, Tourdias *et al*. [2] have recently shown in a model of focal inflammation that AQP4 upregulation was associated with early edema formation via increased ADC, peak BBB disruption, and increased pro-inflammatory cytokine secretion. Diffusion tensor imaging (DTI) takes into account the non-uniform directionality of water flow (anisotropy) in the brain. This anisotropy has mainly been attributed to myelinated neuronal axons in the white matter tract, but recent evidence has hinted towards the possible role of astrocytes and glial scars in DTI signal changes after traumatic brain injury [73]. In fact, increased anisotropy was correlated with reactive astrocytes and not with axonal changes in the perilesional cortex [73]. This idea is supported by a study showing a correlation with changes in DTI signals associated with hypertrophic astrocytes and increase of AQP4 [74]. As AQP4 expression changes after brain injury in astrocytes and microglia, it is rational to think that MRI may be a useful tool to evaluate the evolution of the neuroinflammatory process, especially in conjunction with AQP4 and edema.

### Edema resolution in inflammatory conditions and AQP4

In focal brain inflammation, AQP4 expression is upregulated during the edema resolution phase at 2 to 14 days post injury [2]. However the exact role of this increase in AQP4 is still a matter of discussion. In brain injection of l-α-lysophosphatidylcholine, a significant increase in AQP4 expression was observed at the edema resolution phase (7, 14, and 20 days post injection) compared to the edema build-up phase (1 and 3 days post injection). In this model, the edema resolution phase was defined as a return to baseline for ADC values and a lower IL-1β mRNA level, compared to the edema build-up phase [2]. Interestingly, a similar observation was made in a model of juvenile traumatic brain injury with upregulation of AQP4 observed during the edema resolution period when ADC is returning to normal [41]. These data suggest that the presence of AQP4 plays a positive role during edema resolution by facilitating water extravasations from the brain parenchyma to liquid compartments including CSF and blood vessels (Figure 2).

In stroke pathophysiology, animals with a pre-existing inflammatory condition had aggravated stroke outcomes as seen by more edema and BBB damage at 24 h after injury compared to groups with no pre-existing inflammation in the periphery [75]. Pre-existing systemic inflammation induced a surge in the levels of IL-1 in the ischemic cerebral cortex [75]. Interestingly, increase in IL-1α expression bordering dilated blood vessels in the ipsilateral cortex was observed, signifying a possible direct role of pro-inflammatory cytokines on edema formation [75]. Because IL-1β has been shown to induce AQP4 in astrocytes [76,77], and blocking either AQP4 through gene deletion [78] or IL-1β through anti-IL-1β antibody [79] was seen to decrease edema, AQP4 may be a possible target for systemic inflammation leading to increased edema. In a mouse model of atherosclerosis (APOE^−/−^ mice under high fat diet), development of chronic inflammation due to adhesion of a large number of T cells and macrophages in the vasculature [80], as well as microglial activation in the brain is observed [81]. These mice upon aging showed BBB leakage and higher astrogliosis associated with increased AQP4 [4]. These changes may contribute to a worse outcome in aged atherosclerotic patients who suffer an ischemic stroke because of higher risk of edema.

### AQP4 and microglial activation after injury

**Figure 3:**
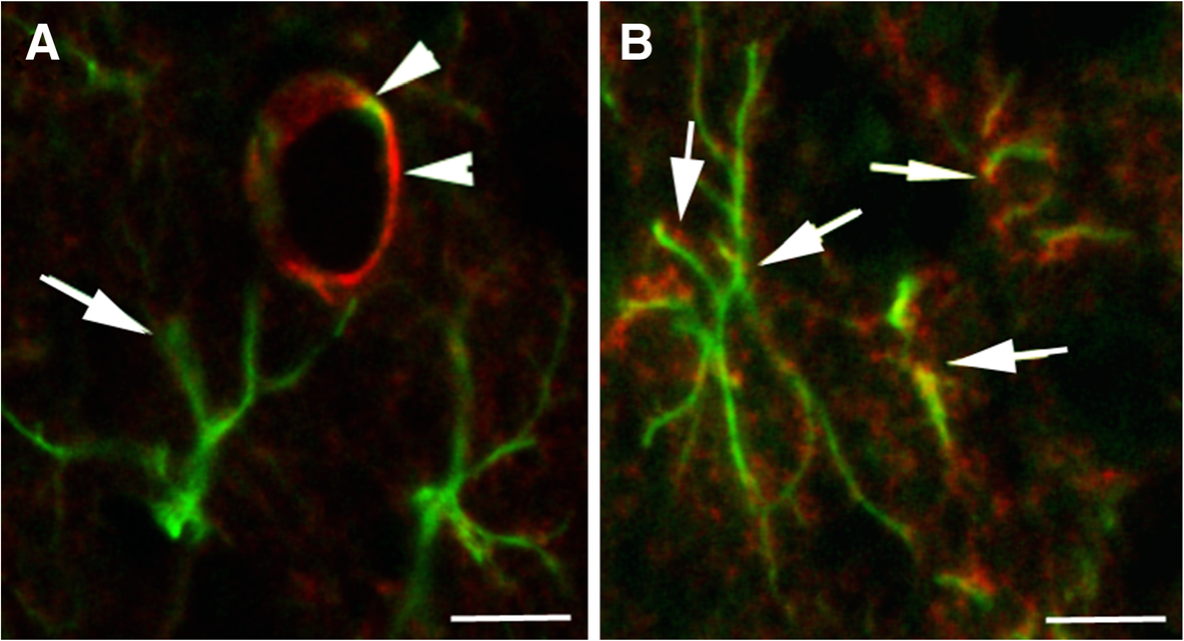
**AQP4 distribution in the astrocyte in normal cortex and after brain injury. **(**A**) Confocal picture of AQP4 immunostaining (red, arrow heads) in normal brain shows the presence of the water channel protein on the astrocyte endfoot (GFAP staining, green, arrow) in contact to the blood vessels in the cortex. (**B**) Confocal pictures of the AQP4 immunostaining (red) on reactive astrocytes revealed with GFAP immunolabelling (green) in the cortex after traumatic brain injury. The presence of the AQP4 staining is not only localized on the endfeet in contact to the blood vessels but also distributed in all astrocyte processes (arrows). Scale bar 10 μm.

There are recent interesting data concerning the relationship between AQP4 and microglial activation. A link between neuroinflammation and AQP4 was described using the AQP4^−/−^ mice that are more susceptible to seizures (decreased seizure latency and increased seizure severity) compared to WT 1 month after TBI and associated with a decrease in neuroinflammatory processes [82]. This difference is related to the neuroinflammatory response showing less astrogliosis and increased microglial activation in AQP4^−/−^ compared to WT mice. Minocycline injection in AQP4^−/−^ inhibited the increase in microglia and also mitigated the severity of the post-traumatic seizure [82]. Similar observations were reported in a model of cryoinjury with increased microglia and reduced astrogliosis in AQP4^−/−^ mice compared to WT at 7 and 14 days post injury. In this model the authors reported a decrease in the lesion volume and lower neuronal loss in AQP4^−/−^ mice compared to WT at 1 day after injury, and the opposite result at 7 and 14 days [83]. Similarly, in adult rats, intravenous minocycline administration after TBI [84] and subarachnoid hemorrhage [85] resulted in less BBB disruption associated with decreased MMP9 and AQP4 at 1 day post injury. In our lab, we have also observed that treatment with small interference RNA (siRNA) targeted against AQP4 (siAQP4) after juvenile TBI showed a decrease in AQP4 associated with less BBB disruption, edema, more NeuN positive cells, and better behavior outcomes compared to the control group at 3 days post injury (unpublished data). As shown in the adult model, we have also noticed a significant increase of activated microglia cells and decreased astrogliosis around the lesion at 3 days post injury in siAQP4-treated rats compared to controls (unpublished data). All together, these data underline that changes in AQP4 expression are associated with changes in astrogliosis and microglia activation in acute brain injury (Figure 3). Astrogliosis may require the presence of AQP4 to facilitate the water movement necessary for the migration [29,40] and hypertrophy. However, the mechanism behind the decrease of the AQP4 and activation of microglia is less obvious and still unknown. One possible mechanism behind the changes observed in post-traumatic or ischemic microglia activation and cytokine release in response to AQP4 downregulation or inhibition may be partly due to the presence of stretch-activated Cl^-^ channels expressed in microglia [86,87]. Stretch-activated/swelling-activated Cl^-^ channels are activated by osmotic stress [88]. It has been observed that the activation of these channels contributes the maintenance of the non-activated (ramified) phenotype of microglia [86]. Because AQP4 is responsible for water transport, inhibition of AQP4 either through genetic deletion or siRNA will alter the osmotic stress within the extracellular space surrounding the microglia, changing the activation status of the swelling activated chloride channels, resulting in microglial activation (Figures 2 and 3). Another possibility lies in the cross-talk that occurs between astrogliosis and microglial activation [34]. It is possible that the decreased extent of injury-induced reactive astrogliosis as a result of knocking down AQP4 caused increased microglial activity.

In summary, the presence of AQP4 seems to play a detrimental role acutely, but at a later phase starting from around 7 days post injury for at least 1 month, AQP4 may play a beneficial role that seems to be involved with inhibiting activation of microglia and promoting edema resolution.

## Conclusion

As reviewed, AQP4 has a key role in the edema process, which may be followed by ADC changes in different brain pathologies. However, whether the presence of AQP4 is beneficial or detrimental seem to depend on the timepoint and injury models. In vasogenic edema, where the BBB is compromised, AQP4 seems to play a beneficial role in eliminating accumulating water from the extracellular space of the CNS (Figure 2). Edema is frequently associated with neuroinflammation with activation of the microglia and astrogliosis. The upregulation of AQP4 previously associated with edema recovery may also contribute to the neuroinflammatory process in astrogliosis and microglia inactivation. In fact, absence or decrease of AQP4 is associated with decrease of astrogliosis and increase of microglia activation. However, the molecular mechanisms of the water channel, AQP4, with the inflammation process are still unknown. But these recent data are encouraging to hypothesize that AQP4 could be a common denominator between edema and neuroinflammation, and underscores the importance of independent investigation to understand how AQP4 is contributing to the neuroinflammatory and edematous process.

## Competing interests

The authors declare that they have no competing interest.

## Authors’ contributions

AF and JB wrote and edited the manuscript. All authors have read and approved the final manuscript.
